# High-altitude medicine

**DOI:** 10.4103/0019-5278.64608

**Published:** 2010

**Authors:** Swapnil J. Paralikar, Jagdish H. Paralikar

**Affiliations:** Department of Physiology, Medical College, Baroda, India; 1 Occupational Health Consultant, Baroda Textile Effects Limited, Baroda, India

**Keywords:** Acclimatization, acute mountain sickness, high altitude, high-altitude cerebral edema, high-altitude illnesses, high-altitude pulmonary edema

## Abstract

Sojourns to high altitude have become common for recreation and adventure purposes. In most individuals, gradual ascent to a high altitude leads to a series of adaptive changes in the body, termed as acclimatization. These include changes in the respiratory, cardiovascular, hematologic systems and cellular adaptations that enhance oxygen delivery to the tissues and augment oxygen uptake. Thus there is an increase in pulmonary ventilation, increase in diffusing capacity in the lung, an increase in the cardiac output and increase in the red blood cell count due to an increase in erythropoietin secretion by the kidney, all of which enhance oxygen delivery to the cells. Cellular changes like increase in the number of mitochondria and augmentation of cytochrome oxidase systems take months or years to develop. Too rapid an ascent or inability to acclimatize leads to high-altitude illnesses. These include acute mountain sickness (AMS), high-altitude cerebral edema (HACE) and high-altitude pulmonary edema (HAPE). Acute mountain sickness is self limiting if recognized early. Both HACE and HAPE are life threatening and need to be treated aggressively. The key to treatment of these illnesses is early recognition; administration of supplemental oxygen; and descent if required. Drugs like acetazolamide, dexamethasone, nifedipine may be administered as recommended.

## INTRODUCTION

Each year, millions of people travel to high altitudes, viz., above 1500 m, visiting recreation areas in the Himalayas in Asia, Alps in Europe, Rockies in the United States and Andes in South America. In addition, every year thousands of adventurers scale the highest peaks in the world, testing the limits of human endurance. Medical scientists attempt to conquer the unique challenges posed by ascent to a higher altitude. This article reviews the basic physiology of ascent to high altitude, as well as the pathophysiology, recognition and management of medical problems associated with high altitude.

## HISTORY OF HIGH-ALTITUDE MEDICINE

In 1920, the British physiologist Alexander M. Kellas predicted that "Mount Everest could be ascended by a man of excellent physical and mental constitution in first-rate training, without adventitious aids if the difficulties are not too great."[1] Yet the summit was reached only 33 years later, in 1953, by Sir Edmund Hillary and Tenzing Norgay; and in 1978, by Reinhold Messner and Peter Habeler, without supplemental oxygen — achievements at the body's outer limits.

That high altitude can have deleterious effects on the body has long been recognized. Perhaps the first reference to illness associated with altitude was recorded nearly 2000 years ago by Tseen Hanshoo, who described the "Great and Little Headache" mountains on the journey along the Silk Road.[2] A description of a monk foaming at the mouth during the ascent of a mountain pass in AD 403 is believed to have been a form of high-altitude sickness.[3]

With the advent of hot-air ballooning in the 19^th^ century, French and English adventurers described deleterious effects of ascent to high altitude. By the end of the 19^th^ century, Angelo Mosso, an Italian physiologist and climber, had built the advanced Regina Margerita on Monte Rossa in the Italian Alps (4,559 m). In 1960, Charles Houston, an Aspen internist, described noncardiogenic pulmonary edema i.e. high-altitude pulmonary edema (HAPE), in a healthy 21-year-old cross-country skier.[4] The large number of Indian troops stationed in the Himalayas provided further description of HAPE in otherwise healthy young men.[5,6] In 1991, a group of experts met to define criteria for the diagnosis of illnesses associated with high altitude. These are known as Lake Louis Criteria.

## CLASSIFICATION OF HIGH ALTITUDE

### High altitude (1500 to 3500 m)

- The onset of physiologic effects of diminished inspiratory oxygen pressure (PIO_2_) includes decreased exercise performance and increased ventilation (lowering of arterial PaCO_2_).
- Minor impairment exists in arterial oxygen transport (arterial oxygen saturation [SaO_2_] at least 90%, but arterial PO_2_ is significantly diminished).
- Because of the large number of people who ascend rapidly to 2500 to 3500 m, high-altitude illness is common in this range.

### Very high altitude (3500 to 5500 m)

- Maximum SaO_2_ falls below 90% as the arterial PO_2_ falls below 60 mm Hg.
- Extreme hypoxemia may occur during exercise, during sleep and in the presence of high-altitude pulmonary edema or other acute lung conditions.
- Severe altitude illness commonly occurs in this range.

### Extreme altitude (above 5500 m)

- Marked hypoxemia, hypocapnia and alkalosis are characteristic of extreme altitudes.
- Progressive impairment of physiologic function eventually outstrips acclimatization. As a result, no human habitation occurs above 5500 m.[7]

## ENVIRONMENT AT HIGH ALTITUDE

**Figure 1:**
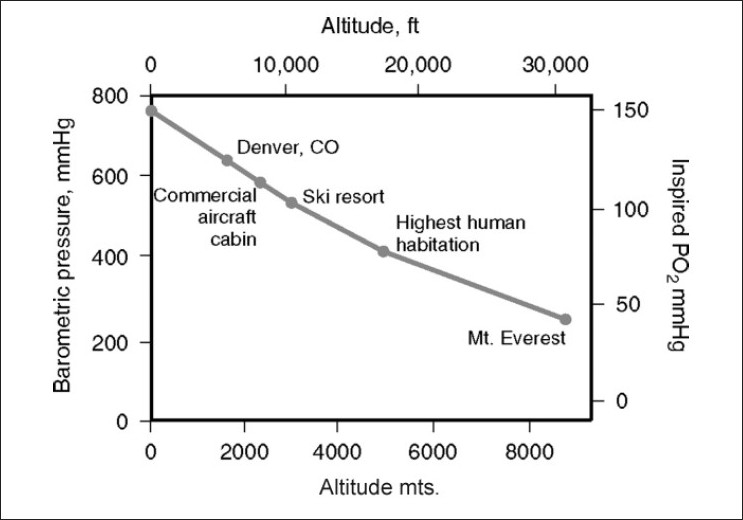
Relationship between altitude, barometric pressure and inspired PO_2_

Barometric pressure falls with increasing altitude in a logarithmic fashion. Therefore, the partial pressure of oxygen (21% of barometric pressure) also decreases, resulting in the primary insult of high altitude: hypoxia [Figure 1]. At approximately 5800 m, barometric pressure is one half that of sea level; and on the summit of Mt. Everest (8848 m), the barometric pressure is 253 mm Hg. Consequently, the alveolar PO_2_ is only about 35 mm Hg. (Alveolar PO_2_ at sea level is 104 mm Hg.)[7,8]

The relationship of barometric pressure with altitude changes with the distance from the equator. Thus polar regions afford greater hypoxia at high altitude, in addition to extreme cold. Also, pressure is lower in winter than in summer. Temperature decreases with altitude, and the effects of cold and hypoxia are generally additive in provoking cold-related injuries and high-altitude pulmonary edema.[9,10]

## PHYSIOLOGICAL ADAPTATIONS AT HIGH ALTITUDE—ACCLIMATIZATION

Rapid ascent from sea level to high altitudes, viz., greater than 2500 m, can cause a number of disturbances: ranging from mild sickness to life-threatening edema of the brain and lungs. However, even extremely high altitudes can be scaled without supplemental oxygen if the ascent is gradual. The process by which individuals gradually adjust to hypoxia and enhance survival and performance is termed *acclimatization*. These integrated responses improve oxygen delivery to the cells through adjustments in the respiratory, cardiovascular and hematologic systems and augment the cellular oxygen uptake and utilization mechanisms.

## VENTILATION

Exposure to hypoxia at high altitude increases ventilation by stimulating the peripheral chemoreceptors. This lowers alveolar carbon dioxide, increases alveolar oxygen and improves oxygen delivery. This response starts at an altitude as low as 1500 m, and within the first few minutes to hours of high-altitude exposure. The carotid body, sensing a decrease in arterial PO_2_, signals the central respiratory center in the medulla to increase ventilation. This carotid body function [hypoxic ventilatory response (HVR)] is genetically determined but is influenced by a number of extrinsic factors. General metabolic stimulants, such as caffeine and cocoa; and respiratory stimulants, such as progesterone, increase the HVR.[11] On the other hand, respiratory depressants, such as alcohol and fragmented sleep, depress the HVR. Physical conditioning apparently has no effect on the HVR. Numerous studies have shown that a good ventilatory response enhances acclimatization and performance and that a very low HVR may contribute to illness. However, over a normal range of values, the HVR is not a reliable predictor of susceptibility to altitude illness.[7]

The immediate increase in ventilation blows off large quantities of carbon dioxide, producing hypocapnia and alkalosis. This inhibits the central respiratory center, causing a 'braking' effect on ventilation. The kidney compensates for the alkalosis by excreting the excess bicarbonate and conserving hydrogen ions. This metabolic compensation for the respiratory alkalosis restores the blood and cerebrospinalfluid (CSF) pH, increasing ventilation. Ventilation reaches maximum only 4-7 days after residence at the same altitude. The plasma bicarbonate concentration continues to drop and ventilation continues to increase with each successive increase in altitude.[8]

## CIRCULATION

### Systemic circulation

Increased sympathetic activity on ascent causes an initial mild increase in blood pressure, a moderate increase in heart rate and cardiac output and an increase in venous tone. Stroke volume is low because of decreased plasma volume as a result of the bicarbonate diuresis, a fluid shift from the intravascular space and suppression of aldosterone. Resting heart rate returns to near sea level values with acclimatization except at extremely high altitudes.

During Operation Everest II (OE II), cardiac function was appropriate for the level of work performed, and cardiac output was not a limiting factor for performance.[12] Interestingly, myocardial ischemia at high altitude has not been reported in healthy persons, despite extreme hypoxemia. This is partly due to reduction in myocardial oxygen demand from reduced maximal heart rate and cardiac output.

Pulmonary capillary wedge pressure is low, and catheter studies have shown no evidence of left ventricular dysfunction or abnormal filling pressures in humans at rest.[13] On echocardiography, the left ventricle is smaller than normal because of decreased stroke volume, whereas the right ventricle may become enlarged.[14] The increase in pulmonary artery pressure because of pulmonary vasoconstriction does not cause left ventricular diastolic dysfunction because of compensatory increased atrial contraction.[15]

### Pulmonary circulation

Hypoxia causes hypoxic pulmonary vasoconstriction (HPV); this increases the pulmonary arterial pressure, causing pulmonary hypertension. Mild pulmonary hypertension is augmented by exercise, with values reaching near systemic levels,[16] especially in individuals with previous history of high-altitude pulmonary edema (HAPE).[17,18] During OE II, Groves and colleagues[16] demonstrated that even when associated with a mean pulmonary artery pressure of 60 mm Hg, cardiac output remained appropriate and right atrial pressure did not rise above sea level values. Thus right ventricular function was intact in spite of extreme hypoxemia and pulmonary hypertension.

### Cerebral circulation

Cerebral oxygen delivery is the product of arterial oxygen content and cerebral blood flow (CBF) and depends on the net balance between hypoxic vasodilation and hypocapnia-induced vasoconstriction. CBF increases, despite the hypocapnia, when PaO _2_  is less than 60 mm Hg (altitude greater than 2800 m). Recent studies have shown individual variations and also impairment in cerebral autoregulation. The individual variation in cerebral blood flow is linked to individual variation in the ventilatory response to hypoxia.[19]

## BLOOD

### Hemopoeitic response to altitude

Ever since the observation in 1890 by Viault[20] that hemoglobin concentration was higher than normal in animals living in the Andes, scientists have regarded the hemopoeitic response as an important part of the acclimatization process.

Hypoxia is the main stimulus for the release of the hormone erythropoietin from the kidney. Erythropoietin acts on the erythropoietin-sensitive committed stem cells in the bone marrow to stimulate red blood cell production.[21] The hormone is detectable within 2 hours of ascent, nucleated immature red blood cells can be found on peripheral smear within days, and new red blood cells are in circulation within 4 to 5 days.

Initially, the hemoglobin concentration increases as a result of a decrease in plasma volume due to the diuresis. This may impair exercise performance. Longer-term acclimatization leads to an increase in blood volume as well as red blood cell mass, thereby increasing blood volume.[7]

### Oxygen-hemoglobin dissociation curve

The respiratory alkalosis produced by the hyperventilation shifts the oxygen-hemoglobin dissociation curve to the left; but a concomitant increase in red cell 2,3 Diphosphoglycerate (2-3, DPG) tends to shift the curve to the right. A net result is a small increase in P_50_ (i.e. the partial pressure of oxygen at which hemoglobin is 50% saturated with oxygen). The resultant decrease in affinity of hemoglobin for oxygen makes more O_2_ available to the tissues.[21]

### Tissue changes

Compensatory changes also occur in the tissues. Hypoxia causes hypoxia-inducible factor-1α to stimulate vascular endothelial growth factor (VEGF) production. This promotes angiogenesis, augmenting blood flow and supplying more oxygen to the tissues.[22] The mitochondria, which are the sites of oxidative reactions, increase in number, and myoglobin increases, which facilitates movement of O_2_ into the tissues. The tissue content of cytochrome oxidase also increases.[21]

### Sleep at high altitudes

Disturbed sleep is common at high altitudes, with nearly all subjects complaining of a disturbed sleep. At moderate altitude, sleep architecture is changed with reduction in stage 3 and 4 sleep, increases in stage 1 time and little change in stage 2. Overall, there is a shift from a deeper to a lighter sleep. In addition, more time is spent awake, with significantly increased arousals. Disturbed sleep architecture may be related to periodic breathing. Sleep quality has little relation to susceptibility to high-altitude illnesses.

## HIGH ALTITUDE ILLNESSES

Syndromes at high altitude are attributable to hypobaric hypoxia. High-altitude illness is a collective term for a cluster of acute clinical syndromes that are a direct consequence of rapid ascent to high altitude, viz., above 2500 m.[23] The acute syndromes affecting the brain include acute mountain sickness (AMS) and high-altitude cerebral edema (HACE). The acute syndrome affecting the lung is high-altitude pulmonary edema (HAPE). All unacclimatized sojourners to high altitude are potentially at risk. The characteristic cerebral or pulmonary abnormalities are not subtle, but when unrecognized or ignored, they may progress to death.

## ACUTE MOUNTAIN SICKNESS

### Clinical presentation

Acute mountain sickness (AMS) is defined as headache in the setting of recent altitude gain and typical symptoms, which include anorexia, nausea, vomiting, insomnia, dizziness or fatigue.[24] The generally accepted Lake Louis Scoring System requires the presence of headache and at least one of the other symptoms, rated in a scale of 1 to 3.[24] Headache is the cardinal symptom; it is bitemporal, throbbing, worse during the night and on awakening. These initial symptoms are strikingly similar to an alcohol hangover.

Specific physical signs are lacking. Heart rate is variable, blood pressure is normal and pulse oximetry is of limited diagnostic value. Absence of the normal high-altitude diuresis, evidenced by lack of increased urine output and retention of fluid, is an early finding in AMS, though not always present.

Given the nonspecific nature of the symptoms, AMS is commonly confused with other conditions like viral flu–like illness, hangover, exhaustion, medication or drug effect. However, a trial of oxygen breathing or descent can help to discriminate these other conditions from AMS.

### Pathophysiological mechanisms

The pathophysiological mechanisms of mild-to-moderate AMS differ from those of severe AMS. Mild-to-moderate AMS is characterized by hypoventilation, impaired gas exchange (interstitial edema), fluid retention and redistribution, and increased sympathetic drive. Persons with a low hypoxic ventilatory response (HVR) are more likely to suffer AMS than those with a high ventilatory drive. The mechanism of fluid retention may be multifactorial. Elevated levels of antidiuretic hormone, activation of the renin-angiotensin-aldosterone system and an enhanced sympathetic drive contribute to fluid retention by the kidneys.[25] Moderate-to-severe AMS is associated with white matter edema in the brain. The most compelling evidence in this matter was provided by the study by Hackett and colleagues.[26] These data suggest that the edema is vasogenic in origin with an increase in permeability of the endothelium. The cause of the leak may be increase in intravascular pressure or hypoxemia *per se*.

### Treatment

Early diagnosis is the key in the treatment of acute mountain sickness because treatment is easier and more successful in the early stages. Mild AMS can be treated by halting the descent and waiting for acclimatization to improve, which may take 3 to 4 days. Acetazolamide, a carbonic anhydrase inhibitor, should be given in the dose of 250 mg twice a day for 3-5 days. It aids and speeds up acclimatization. Symptomatic treatment can be given with analgesics (aspirin, ibuprofen or other NSAIDs) for headache. Promethazine (25-50 mg) is useful for the treatment of nausea and vomiting.

Steroids, particularly dexamethasone, are effective in relieving symptoms. Dexamethasone was used by Hackett and colleagues[27] in the dose of 4 mg orally or intramuscularly. Its mechanism of action is not known. Because symptoms recur after stopping dexamethasone, it is thought not to aid acclimatization. Hence it should be given in conjunction with acetazolamide.

Descent to an altitude lower than where symptoms began effectively reverses AMS. Descending 500 to 1000 m is usually sufficient. Exertion should be minimized. Oxygen, if available, is particularly effective. Hyperbaric chambers, which simulate descent, have been used to treat AMS and aid acclimatization. They are effective and require no supplemental oxygen.[7]

### Prevention

Prevention of all altitude illnesses requires ascent at a gradual rate allowing time for acclimatization. A general guideline is that at altitudes greater than 3000 m, one should not spend subsequent nights 300 m higher than the previous night. A rest day is recommended every 2 to 3 days. Anyone with the symptoms of AMS should not ascend until the symptoms are improved.

The clinician should evaluate patients who are seeking advice on trips to high altitude. A history of high-altitude problems, the altitude profile and the speed of ascent are all important factors. The physician should educate patients about the signs and symptoms of high-altitude illnesses and inform them of the risks assumed by the high-altitude traveler.

### Acetazolamide prophylaxis

Acetazolamide is the drug of choice for prophylaxis against AMS. A carbonic anyhydrase inhibitor, the drug slows the hydration of carbon dioxide:

CO_2_ + H_2_O ↔ H_2_CO_3_ ↔ H^+^ + HCO_3_^-^

[Both these reactions are catalyzed by the enzyme carbonic anhydrase (CA).]

Acetazolamide affects the red blood cells, kidneys, lungs and brain. Being a renal CA inhibitor, acetazolamide decreases bicarbonate reabsorption, causing diuresis. The resultant acidosis effectively stimulates the medullary respiratory center, increasing ventilation and enhancing oxygen delivery to the cells. In addition, acetazolamide inhibits CSF production and CSF pressure and also inhibits nocturnal antidiuretic hormone (ADH) secretion.

Indications for acetazolamide prophylaxis include rapid ascent (in 1 day or less) to altitudes greater than 3000 m; a rapid gain in sleeping altitude, for example, moving camp from 4000 to 5000 m; and a past history of AMS or HAPE.

Side effects include paresthesias, polyuria; and less commonly, nausea, drowsiness, impotence and myopia. Because it impairs the hydration of carbon dioxide on the tongue, it allows carbon dioxide to be tasted and can ruin the flavor of carbonated beverages, including beer. It should be given with caution in patients with allergies to sulfa drugs, and is contraindicated in the rare case of allergy to acetazolamide itself.[7]

## HIGH-ALTITUDE CEREBRAL EDEMA

High-altitude cerebral edema (HACE) is more likely a continuum of AMS. HACE also occurs in 13% to 20% of individuals with HAPE and in about 50% of individuals who die from HAPE.[28] According to the Lake Louis consensus definitions, HACE can be diagnosed if ataxia occurs in a person with acute mountain sickness, or both ataxia and mental status changes occur in the absence of AMS [Table 1]. Mental status changes include confusion, impaired mentation, drowsiness, stupor and coma. Pulse oximetry in a patient with HACE reveals exaggerated hypoxemia. Radiologic findings may reveal concomitant pulmonary edema.

**Table 1:** Lake Louis Consensus definitions of high altitude[29]

Acute mountain sickness (AMS)
- In the setting of a recent gain in altitude, there is presence of headache and at least one of the following:
- Gastrointestinal (anorexia, nausea or vomiting)
- Fatigue or weakness
- Dizziness or lightheadedness
- Difficulty sleeping
High-altitude cerebral edema (HACE)
- Can be considered "end stage" or severe AMS. In the setting of a recent gain in altitude, there is either —
- the presence of a change in mental status and/ or ataxia in a person with AMS
- or the presence of both mental status changes and ataxia in a person without AMS.
High-altitude pulmonary edema (HAPE)
In the presence of a recent gain in altitude, the presence of the following:
At least two of the following symptoms —
- Dyspnea at rest
- Cough
- Weakness or decreased exercise performance
- Chest tightness or congestion
At least two of the following signs:
- Crackles or wheezing in at least one lung field
- Central cyanosis
- Tachypnea
- Tachycardia

HACE is a medical emergency and should be treated aggressively. Immediate descent of up to at least 600 m is required. In addition, oxygen should be administered (2-4 L/min), and acetazolamide should be given in the dose of 250 mg bd. Dexamethasone is an excellent drug to treat HACE and is more effective in higher doses (8 to 10 mg IM, IV or po initially, and 4 mg q6h). In previously healthy individuals, the side effects of dexamethasone are inconsequential, and if the drug is started at the onset of symptoms of HACE while descent is undertaken, the drug can be life saving.[28,29]

## HIGH-ALTITUDE PULMONARY EDEMA

### Clinical presentation

High-altitude cerebral edema (HACE) is the most common cause of death related to high altitude. It is a noncardiogenic form of edema, which typically occurs at altitudes above 3000 m, affecting previously healthy individuals who ascend rapidly from sea level and may not have suffered HAPE even with repeated altitude exposure. HAPE usually occurs within the first 2 to 4 days of ascent to higher altitudes, most commonly on the second night. HAPE begins with a subtle nonproductive cough and shortness of breath, both at rest and especially with attempts to exercise modestly. It progresses to a debilitating degree of dyspnea, even at rest, and cough productive of pink, frothy sputum. Tachypnea and tachycardia are present; pulse oximetry reveals marked hypoxemia. Imaging of the thorax reveals patchy opacities with inconsistent predominance of location, but often infiltrates are seen initially in the region of the right middle lobe.[30]

### Pathophysiological mechanisms

Studies have shown three main pathophysiological mechanisms responsible for HAPE: pulmonary hypertension, 'stress failure' of the pulmonary capillaries and disturbed alveolar fluid clearance.

#### Pulmonary hypertension

Hypoxia causes hypoxic pulmonary vasoconstriction (HPV). This vasoconstriction is nonhomogeneous (uneven) because of differences in reactivity of smooth muscle in different parts of the lung to hypoxia; or due to different anatomic characteristics, such as distribution of muscularized arterioles. This leads to increased pressure and flow in the perfused areas, causing pulmonary hypertension and subsequent edema.[7,21]

#### Stress failure of the pulmonary capillaries

The pulmonary edema is of high-permeability type, with leakage of proteins and white blood cells. This prompted West to hypothesize that edema occurs due to 'stress failure' of the pulmonary capillaries.[31]

#### Disturbed alveolar fluid clearance

Alveolar fluid balance is maintained by transport of alveolar fluid across the epithelium and its drainage by lymphatics. Hypoxia inhibits the activity of apical epithelial sodium channels (ENaCs) and of the basolateral sodium-potassium ATPase pumps. Water reabsorption is coupled to that of sodium, causing fluid to accumulate in the alveoli.[32]

### HAPE susceptibility

HAPE-susceptible individuals (HAPE-s) show at sea level an abnormal rise of pulmonary artery pressure and pulmonary vascular resistance during hypoxic challenge at rest and during exercise. Also HAPE-s display an overactivity of the sympathetic nervous system and impaired endothelial function. The latter is evidenced by reduced nitric oxide synthesis during hypoxia, and elevated levels of endothelin, a potent pulmonary vasoconstrictor.[7]

### Treatment

Early recognition is the key to successful outcome, as in other high-altitude illnesses. Immediate descent, of up to 500 to 1000 m is required. Oxygen, if available, should be administered immediately. Oxygen increases arterial oxygenation and reduces pulmonary artery pressure, heart rate, respiratory rate and other symptoms.

Drugs are of limited necessity in HAPE. The calcium channel blocker nifedipine (30 mg slow release every 12 to 24 hours or 10 mg orally repeated as necessary) gives good results. Nifedipine reduces pulmonary artery pressure and slightly improves arterial oxygenation.[33] Glucocorticoids have also been found to be helpful in treating HAPE. They may act by blocking the leak in the capillary layer, or by increasing the activity of the basolateral Na^+^K^+^ ATPase pump.[34] In addition, nitric oxide and PDE-5 inhibitors (sildenafil and tadanafil) have been tested, but further research is needed to clarify their role.

### Prevention

Preventive measures described for AMS also apply to HAPE: graded ascent, time for acclimatization, low sleeping altitudes and avoidance of alcohol and sleeping pills. Exertion may contribute to the onset of HAPE, especially at moderate altitude. Prudence dictates not overexerting for the first 2 days at altitude.

Recent studies show that acetazolamide, by blocking pulmonary vasoconstriction, may be helpful in preventing HAPE.[29] Nifedipine (20 mg slow release every 8 hours) prevented HAPE in those with a previous history of episode.[33]

The drug should be carried by such individuals and started at the first signs of HAPE, or before starting an abrupt ascent. In addition, steroids (dexamethasone), PDE-5 inhibitors (sildenafil and tadanafil) may also be used.

### Re-entry HAPE

Re-entry HAPE occurs in high-landers who return from a trip to low altitude. It is most commonly observed in the Peruvian Andes, where high-altitude residents can return from sea level to high altitude quite rapidly. Researchers have postulated that the increased muscularization of the pulmonary arterioles that develops with chronic high-altitude exposure generates an inordinately high pulmonary artery pressure on re-ascent, causing the edema.
